# The effect of linguistic and visual salience in visual world studies

**DOI:** 10.3389/fpsyg.2014.00176

**Published:** 2014-03-04

**Authors:** Federica Cavicchio, David Melcher, Massimo Poesio

**Affiliations:** ^1^Center for Mind/Brain Sciences, Università di TrentoRovereto, Italy; ^2^School of Psychology, University of BirminghamBirmingham, UK; ^3^School for Computer Science and Electronic Engineering, University of EssexEssex, UK

**Keywords:** linguistic salience, visual salience, visual world paradigm, centering theory, saliency map

## Abstract

Research using the visual world paradigm has demonstrated that visual input has a rapid effect on language interpretation tasks such as reference resolution and, conversely, that linguistic material—including verbs, prepositions and adjectives—can influence fixations to potential referents. More recent research has started to explore how this effect of linguistic input on fixations is mediated by properties of the visual stimulus, in particular by visual salience. In the present study we further explored the role of salience in the visual world paradigm manipulating language-driven salience and visual salience. Specifically, we tested how linguistic salience (i.e., the greater accessibility of linguistically introduced entities) and visual salience (bottom-up attention grabbing visual aspects) interact. We recorded participants' eye-movements during a MapTask, asking them to look from landmark to landmark displayed upon a map while hearing direction-giving instructions. The landmarks were of comparable size and color, except in the Visual Salience condition, in which one landmark had been made more visually salient. In the Linguistic Salience conditions, the instructions included references to an object not on the map. Response times and fixations were recorded. Visual Salience influenced the time course of fixations at both the beginning and the end of the trial but did not show a significant effect on response times. Linguistic Salience reduced response times and increased fixations to landmarks when they were associated to a Linguistic Salient entity not present itself on the map. When the target landmark was both visually and linguistically salient, it was fixated longer, but fixations were quicker when the target item was linguistically salient only. Our results suggest that the two types of salience work in parallel and that linguistic salience affects fixations even when the entity is not visually present.

## Introduction

In the basic set-up of a visual world experiment participants hear a word or an utterance while looking at an experimental display on the screen. Their eye movements are recorded as they listen to the sentences or words. Studies using the visual world paradigm (Cooper, 1974; Tanenhaus et al., 1995) have shown that visual and linguistic information are rapidly integrated when processing spoken instructions in the context of a task-relevant visual world (for a recent review, see Huettig et al., 2011a). These studies showed that participants focus their attention on the target object after hearing the beginning of the target word (see for example Allopenna et al., 1998; Altmann and Kamide, 1999). Chambers et al. (2002) and Wolter et al. (2011) also showed that linguistic input other than referring expressions, such as verbs or scalar references, immediately restrict fixations to the objects that could be the argument of those verbs. For example, when presented with the instruction “Put the cube inside the can,” participants restricted their visual attention to containers of the right size immediately after hearing the word inside.

However, the guidance of saccadic eye movements is a complex process that depends on a multitude of factors, including the (bottom-up) visual salience of the item presented on the screen (see, e.g., Itti and Koch, 2000; Schubö, 2009; Tatler et al., 2011). Such studies have led to the development of a saliency map model of the integration of such bottom-up factors (Koch and Ullman, 1985; Itti and Koch, 2001). A saliency map is an explicit two dimensions map based on early visual processing. It provides a control strategy in which the focus of attention scans the saliency of an image or a complex scene in order of decreasing saliency. A saliency map is presented as an image depicting stimulus saliency at each location in the visual scene.

Although theories developed on the basis of the visual world paradigm have tended to be underspecified with respect to how the bottom-up visual salience of an object affects the integration of linguistic and visual information, more recently the study of such factors has become the focus of research (e.g., see Huettig et al., 2011a and the other papers in that issue, in particular Salverda and Altmann, 2011; Salverda et al., 2011). On the basis of evidence such as reported by Ballard et al. (1995), it has been shown by Salverda et al. (2011) that task salience overrides bottom-up visual salience. The pattern of saccades and fixations during a scene scan is primarily governed by bottom up visual salience, but as the task requires participants to derive information from the visual input, visual salience alone turns out to be a poor predictor of gaze patterns. But other issues still need research. One such issue is how bottom up visual salience interacts with linguistic salience, which has been shown to strongly affect reference resolution.

It has been repeatedly demonstrated that linguistically introduced entities have different degrees of accessibility (Grosz, 1977; Sanford and Garrod, 1981; Gernsbacher, 1989; Gordon et al., 1993; Gundel et al., 1993; Grosz et al., 1995; Arnold et al., 2000; Brown-Schmidt et al., 2005). Such accessibility, which translates, e.g., in reduced response times for sentences containing references to entities with greater accessibility, is modulated by factors including order of mention (Gernsbacher, 1989; Gordon et al., 1993; Arnold et al., 2000), grammatical function (Gordon et al., 1993; Hudson-D'Zmura and Tanenhaus, 1998), type of Noun Phrase (NP) used to introduce the entity. Entities introduced using proper names are more salient than entities introduced using definites or indefinites (Sanford et al., 1988), repetition (Gordon et al., 1993; Brennan, 1995; Arnold, 1998; Van Gompel and Majid, 2004) and possibly scenario knowledge (Sanford and Garrod, 1981). We illustrate this with the example below, which consists of instructions verbally communicated to participants while they viewed the map of a town.

(1) a. Today we will visit some locations strongly associated with the life of the painter and graphical designer Fortunato Depero.
b. Let us start from the vineyards of the Fedrigotti family that commissioned a lot of work from him.
c. Next we will visit the house where the artist was born.
d. We will end the tour at the Depero Museum.
d′. We will end the tour at the station.

In this example, the abovementioned studies would suggest that in (1), the individual Fortunato Depero, mentioned using a proper name and in salient position in (1a), and then repeatedly mentioned in the subsequent sentences, will be linguistically salient after (1c). Therefore a continuation sentence mentioning Depero, like (1d), will be processed more easily than a continuation sentence that does not contain a reference to that entity, like (1d'). This greater salience will translate in a reduced response time.

The evidence just discussed led to the development of models of linguistic salience stipulating attentional structures that play a role in linguistic salience—similar to the role played by saliency maps with regard to visual salience. The best known among these models, and the most widely used in psycholinguistic research on reference, is the Centering Theory (Gordon et al., 1993; Brennan, 1995; Grosz et al., 1995; Arnold, 1998; Hudson-D'Zmura and Tanenhaus, 1998; Poesio et al., 2004). In the Centering Theory, linguistic salience is viewed as an attentional structure, the Centering Framework (CF) List, which resembles saliency maps in many respects (e.g., in the stipulation of a winner-take-all mechanism leading to a single entity being the most salient at any time) but with some important differences. The CF list is a list of all the entities mentioned in a sentence. The rank of these entities is determined by a combination of the linguistic factors described in the previous paragraphs (order of mention, grammatical function, type of NP, etc.). Visual and task salience factors play no role, and a separate attentional structure called the “focus space stack” is hypothesized for task salience. The entity most likely to be pronominalized in a sentence, the Backward-Looking Center, is defined as the most highly ranked entity in the previous sentence that is still mentioned in the present sentence, i.e., through a combination of grammatical salience and repetition: in (1), Depero is the backward-looking center in sentences b, c, and d; neither a nor d' would have a backward-looking center.

Studies such as Arnold et al. (2000) and Brown-Schmidt et al. (2005) demonstrated that (some of) the factors determining linguistic salience mentioned above (e.g., subjecthood, repetition) affect interpretation in a visual world setting just as they do in sentence response tasks. For instance, when hearing a pronoun, there is a preference for subjects to fixate on an object in the visual world which is linguistically salient. But to our knowledge, no study so far addressed the question of how linguistic salience interacts with visual salience in affecting fixations in a visual world task. This is the general question addressed in this study.

In particular, we were interested in whether there would be interference between visual and linguistic salience in a context in which one landmark is associated to a linguistically salient entity, whereas another landmark is visually salient (bigger or more colorful). The study of eye movements is particularly relevant for this question, since the serial nature of gaze shifts means that the eye can only move to one location at a time. The motor decision of where to move the eye next has been shown to be directly affected by (bottom-up) visual salience, not just task constraints (Gottlieb et al., 1998; Masciocchi et al., 2009; Salverda et al., 2011). It has been suggested that this motor decision reflects activity in a single master salience map which integrates different aspects of attention priority (Gottlieb et al., 1998; Kusunoki et al., 2000; Gottlieb, 2007).

The hypothesis tested here is that in a direction-giving task the landmarks on a map that are associated with linguistically salient entities (although not present on the map) will be the target of more fixations than landmarks not associated with such entities. The interference between linguistic and visual salience—if any—is most likely to be observed in a setting in which the linguistically salient objects are not visually present. In the Arnold et al. (2000) and Brown-Schmidt et al. (2005) studies, the linguistically salient objects were visually present. It could be argued that in such settings there is no need for the subject to create and maintain a separate CF List; whatever attentional structures are used to encode visual salience will suffice. But when the objects mentioned are not visually present the need arises to establish an attentional structure containing linguistically introduced information, such as the CF List. To our knowledge, no previous study has considered this type of setting.

One possibility is that linguistic salience would modulate the activity in this sensorimotor map—i.e., that the CF List proposed in Centering theory coincides with, or at least interacts directly with, the saliency/priority maps proposed in the visual attention literature. Alternatively, linguistic salience might work concurrently with visual salience, influencing the processing of linguistic and visual information through a separate informational structure rather than directly altering the salience map. More specifically, if only one attentional structure is maintained (i.e., if saliency maps and CF List coincide) we would not expect to observe separate effects of linguistic and visual salience. If, on the other hand, the CF List and the saliency map work in parallel, linguistic salience would be expected to affect response (button press) and dwell (fixation durations) times but not saccadic target selection (time to first fixation).

Our research question on the interaction between Linguistic and Visual salience was investigated using four measures. First, to test the effect of CF List we measured response times, as typically done in studies on the effect of linguistic salience on the interpretation of referring expressions (Gernsbacher, 1990; Gordon et al., 1993; Hudson-D'Zmura and Tanenhaus, 1998; Van Gompel and Majid, 2004). Participants were asked to press the mouse button when they identified the sentence visual target (e.g., the train station). To measure linguistic salience, we relied on a finding that goes back to the very first work in the visual world paradigm tradition (Cooper, 1974). Cooper found that subjects would fixate not only on objects explicitly mentioned, but also on objects that were somehow associated with them. In addition to response times and fixations, in order to determine visual salience we analyzed fixation patterns focusing on time to first fixation and fixation dwell on each landmark. Visual salience has been shown to increase saccadic reaction time—we are faster to look at a highly salient target among distractors than at low salience target. Saccadic reaction time (also called fixation speed or time to first fixation) is a process involving target selection and motor execution. It is affected by the ability to find a target among distractors. In contrast, fixation dwell has more to do with how long it takes to fully process a stimulus. In the literature, it is well known that these processes are somewhat independent and are affected by different factors (see for example Platt and Glimcher, 1999; Beutter et al., 2003; Tatler et al., 2011). Fixation dwell is much more complex than saccadic reaction time, involving also linguistic and cognitive factors.

Finally, we used a more realistic scenario to study the interaction of linguistic and visual salience than the simplified settings normally seen in visual world studies—a direction-giving task in a touristic context, in which subjects are looking at landmarks while the guide tells them about their history and the famous people who lived there, such as the artist Fortunato Depero.

## Materials and methods

### Participants

Forty students (18 males, mean age 26.1 years; *SD* 6.4) at the University of Trento, all native speakers of Italian and resident in the Trentino region, took part in the experiment. All participants gave informed consent prior to the start of the experiment. They reported normal or corrected-to-normal vision and normal hearing.

### Materials

Eye movements were recorded using a Tobii x50 eye tracker, with a frame rate of 50 Hz (50 frames per second). Stimuli were presented on a 17-inch TFT monitor at a resolution of 800 × 600. Stimulus presentation was controlled by a PC running E-Prime 1.5 experimentation software. Spoken stimuli were presented binaurally through Sennheiser HD 570 headphones. Response time was collected through mouse button. The mouse was connected to the PC running E-Prime. In this setting the eye tracker sampled the eye position every 24.72 ms.

### Visual stimuli and visual salience manipulation

Visual stimuli consisted of 8 maps with five pictorial landmarks on each of them. There were more landmarks on the maps than there were visual targets as otherwise the last visual target would have been obvious by exclusion. The pictorial landmarks were taken from photographs of natural scenes. The position of the pictorial landmarks was randomized. For example, the first landmark—the train station—could be in any position on the map.

In half of the maps we manipulated visual salience by modifying the size and colors of the last sentence target landmark. The visual salience of each landmark on each map was calculated with Itti-Koch algorithm (Itti et al., 1998) implemented in MATLAB. The purpose of this algorithm is to compute, from an image, a saliency map, modeling a priori the observers' gaze orientation. In Itti et al. (1998) visual salience is determined in a bottom-up fashion by the degree of difference between a spatial location and its spatial surroundings. Position, color, and size were the main variables taken into account to compute the saliency map of landmarks. For each landmark presented on the maps, the algorithm calculated an index from 0 to 1. All the landmarks had a salience index of 0.45 (*SD* = 0.13), ensuring they equally attracted participants' gaze. In visual salience condition, the visually salient landmark had a visual salience index of 1, whereas the other landmarks displayed on the screen at the same time had an average visual salience of 0.45. In total, 4 out of 8 maps had one visually salient landmark (+VS) whereas in the remaining 4 none of the landmarks was visually salient (−VS).

### Linguistic stimuli and linguistic salience manipulation

Eighty sentences were recorded by a male native speaker of Italian (see Appendix). For each map we recorded two different versions: linguistic salience condition (+LS) and no linguistic salience (−LS condition).

In +LS condition, four out of five sentences referred to an item or a person (which we call entity) that was not on the map. For example, the sentence “Today we will visit some interesting location of Depero's artwork” was delivered while the participants attended to a fixation cross. In the following sentences, the word “Depero” was the entity element. To avoid a cognitive load effect, the second sentence, following the fixation cross slide, did not mention the entity element and was the same for all the trials (i.e., “Let's start from the train station”). In both +LS and −LS conditions the last sentence was the same. By the time the last sentence was presented two possible visual targets were left. This was to ensure that participants could not guess what would be the target of the last instruction.

**Figure 1:**
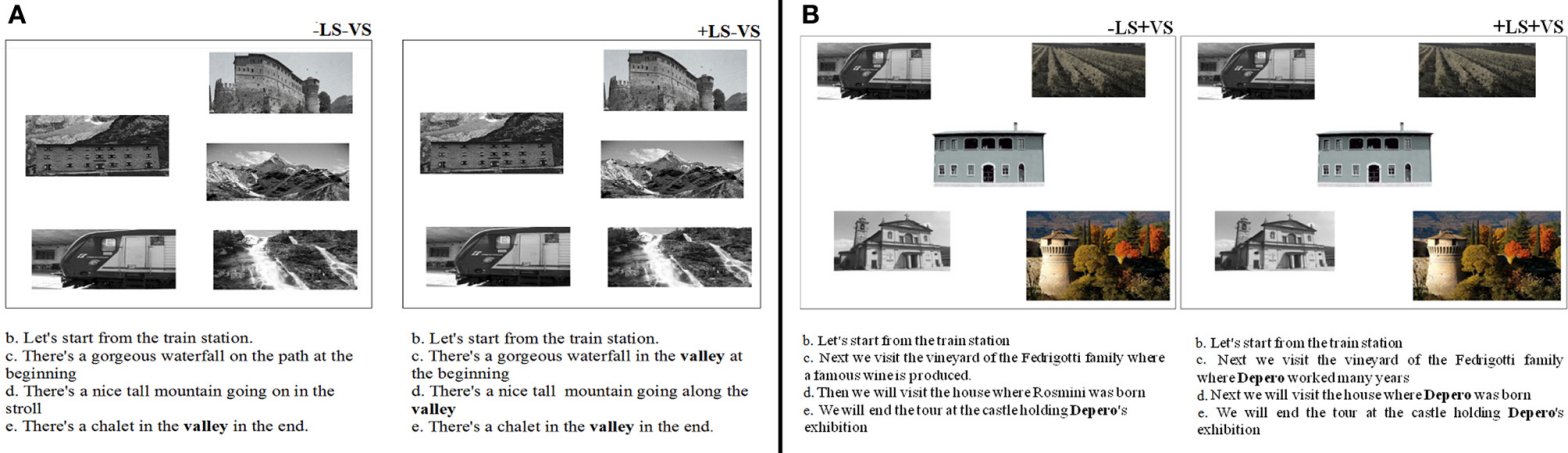
**Example of the experimental stimuli in −LS−VS and +LS−VS (A) and −LS+VS and +LS+VS (B) conditions**. In −LS−VS and +LS−VS conditions the landmarks on the maps had all the same visual salience. In the −LS condition the sentences did not have any entity repeated. In +LS condition an entity (valley) was repeated. The last sentence (e.g. “there's a chalet in the valley in the end”) was the same in both −LS−VS and +LS−VS conditions. In the +VS conditions one of the visual target was visually salient with respect to all the other landmarks displayed on the map at the same time. In the −LS+VS condition the sentences did not have any entity repeated. In the Linguistic Salience condition (+LS+VS) an entity (e.g., the Italian artist Depero) was repeated. The last sentence (e.g., “We will end the tour at the castle holding Depero's exhibition”) was the same in both −LS+VS and +LS+VS conditions.

In half of the trials the correct target was visually salient as well. In −LS condition the same maps used in +LS condition were presented on the screen. However, in −LS condition the sentences did not have any entity repetition. In order to control for complexity, in −LS condition we substituted the entities with words of very similar length and frequency (see Results for more information). In the +LS versions the materials would be as in example (2). In this version, one entity which was not visually present on the map (e.g., Fortunato Depero) was introduced using a proper name in the first sentence while the participants attended a fixation cross. Once the sentence ended, the map appeared. The second phrase (the first in which the participants actually saw the map) prompted the participant to localize the train station. This phrase and the corresponding visual target were identical for all of the conditions (See Figure 1, right panel). Then, the linguistically salient entity was repeatedly mentioned in each new instruction, as in (2).

(2) a. Today we will visit some locations strongly connected with Depero's artwork (fixation cross)
b. Let's start from the train station (visual target = station)
c. next we visit the vineyard of the Fedrigotti family where Depero worked many years (visual target = vineyard)
d. Next we will visit the house where Depero was born (visual target = house)
e. We will end the tour at the castle holding Depero's exhibition (visual target = castle).

In the −LS condition, each phrase referred to different entities instead of always mentioning Depero, as shown in (2′). The final sentence was identical to the condition +LS (see Figure 1, left panel).

(2′) a. Today we will visit some sights in Val Lagarina of great cultural interest. (fixation cross)
b. Let's start from the train station (visual target = station)
c. Next we visit the vineyard of the Fedrigotti family where a famous wine is produced. (visual target = vineyard)
d. Then we will visit the house where Rosmini was born (visual target = house)
e. We will end the tour at the castle holding Depero's exhibition (visual target = castle)

Participants listened to a first sentence (mean duration 3891 ms, *SD* 121 ms) that introduced the task while a fixation cross was shown on the screen. After that, a map appeared and a sentence, the same for all trials and conditions, was read out (“let's start from the train station”—sentence b. in 2 and 2′). The following three sentences had a mean duration of 2493 ms (*SD* 232 ms) and the target word was delivered at 597 ms (*SD* 139 ms). In the Linguistic Salience condition, the last three sentences contained an entity expression, presented for the first time in the first sentence. The entity expression was delivered at 1497 ms (*SD* 98 ms).

### Experimental design

The general design of our experiments follows previous studies using the visual world paradigm. Participants sat in front of a screen while listening to a sentence. Our task was a “look and listen” (Huettig et al., 2011b) version of the Map Task (Anderson et al., 1991) in which participants looked at a map with five pictorial landmarks and were asked by the recorded voice to (mentally) move from one point in the map to the next in response to direction-giving instructions. After the map appeared on the screen, each subject heard a sentence b (2 and 2′).

In the linguistic salience condition, we referred to the target landmark using a description that would associate the object with the linguistically salient entity (e.g., “the vineyard of the Fedrigotti family where Depero worked many years”). In the −LS condition no such associations were provided (e.g., “the vineyards of the Fedrigotti family that produces a famous wine”). Participants saw each of the 8 maps only in one of the two LS conditions. Therefore, they saw each of the 8 maps only once during the experiment. The order of presentation of the 8 maps was counter-balanced across participants.

### Procedure

Participants were seated at approximately 60 cm from the computer screen. The lab was dimly lit. The only two sources of light were the monitor used for stimulus presentation and the monitor of the PC running the eye-tracker. The latter was located behind the participant.

After attending a fixation cross and hearing a first sentence, participants pressed the mouse button and the map appeared on the screen. After that, four sentences followed while a map with five pictorial landmarks was presented on the screen. Participants were asked to press the mouse button when they identified the target landmark mentioned in each of the sentences. They were explicitly told to not move the mouse from landmark to landmark or to click on the landmarks. The next sentence in the sequence was presented only after a button press was made.

### Response times

Response times were recorded from the sentence target onset (e.g., vineyard) by mouse click.

### Measuring fixations

Fixations were recorded throughout the whole experiment. We measured the log odds of fixations on each target over the total number of fixations. We calculated the log odds of fixations because the dependent variable was the region of the screen (target) to which participants directed the gaze at a given moment in time, a variable that is categorical.

As mentioned above, we measured fixations to each sentence target. For example in the sentences “the vineyard where Depero worked” (+LS) vs. “the vineyard where a famous wine is produced”

(−LS) we measured the fixations to the vineyard pictorial image. In visual world studies it is customary to transform the categorical dependent variable “fixation” into a continuous variable by calculating proportions collapsed over time and over trials in the experiment. However, such analysis has recently been criticized because it violates the assumptions that the dependent variable has an unbounded range and that errors are distributed normally and independently of the mean. In addition to that, in the visual world paradigm the dependent variable fixation has multiple observations within item (the target word) and subjects. Therefore, following Barr (2008) and Baayen et al. (2008) we calculated the log odds of fixations for each sentence and compute a linear mixed effect regression with random intercepts and slopes for subject and random intercepts and slopes for item. Following Barr (2008), we calculated for each trial the number of frames in which the participants' gaze fell into the boundaries of the target image. After that, we computed the empirical logs of fixation applying the following formula (McCullagh and Nelder, 1989):

(1)  log[(Y+0.5)/(N−Y+0.5)]

where *Y* is the number of frames in which the participants actually fixated the target object and *N* is the total number of frames in the time window.

Each time window was set from the onset of the target word to the end of the longest sentence. Therefore, the time window was 1000 ms for sentence b and 1750 ms for the other sentences.

Since each of the 40 participants was presented with the 8 maps, we had 320 data points for each of the sentences that were read out.

All analyses on fixations were run in R 2.13.2. The mixed effect models for each sentence were run using function lmer in package lme4_0.999375-39. Our models used the “maximal” random effects justified by the experimental design (Barr et al., 2013): random intercepts by subjects and items, random slopes for linguistic and visual salience by subjects and random slopes for linguistic salience by items. We also calculated the interaction between the fixed factors linguistic and visual salience. As our predictors has two-levels, to standardize the contrast we chose a “deviation coding scheme,” such as (−0.5, 0.5). This coding scheme compares the mean of the dependent variable for a given level (e.g., −LS = −0.5) to the overall mean of the dependent variable. In order to investigate the “effect size” of LS and VS on the dependent variable, we report the Estimates of LS and VS. Finally, to perform hypothesis tests, we used a model comparison approach. For each main effect of interest, we compared the deviance of a full model containing all fixed and random effects to the deviance of a model in which only the fixed effect being tested had been removed. All random slopes and the remaining fixed-effect terms remained in the model. All test statistics followed the chi-squared distribution with one degree of freedom.

## Results

### Response times

**Figure 2:**
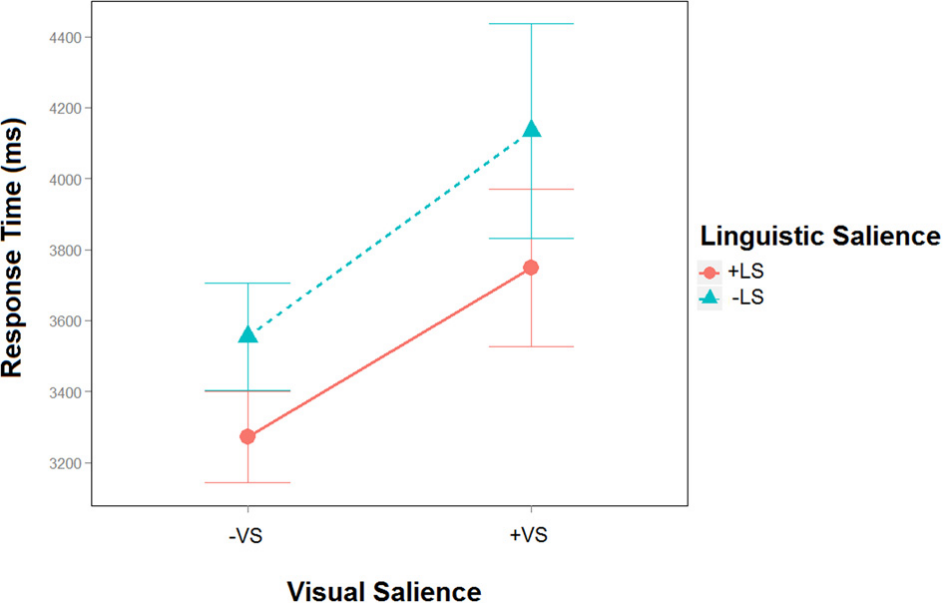
**Response times of the Linguistic (+LS; −LS) and Visual (+VS; −VS) salience in the last sentence**. RTs are significantly faster in the +LS−VS condition compared to the other three conditions whereas RTs are slowest in the −LS+VS compared to all other conditions.

In the last sentence response times varied across the different salience conditions. A linguistic salience × visual salience within subjects ANOVA indicated a significant effect of linguistic salience [*Df* = (1264), *F* = 6.4, *p* = 0.01] and visual salience (*Df* = (1266), *F* = 16.7, *p* < 0.001] but no interaction between the two factors [*Df* = (1264), *F* = 0.96, *p* = 0.3; see Figure 2].

## Fixations

### Sentence B

#### Log odds of fixation

**Figure 3:**
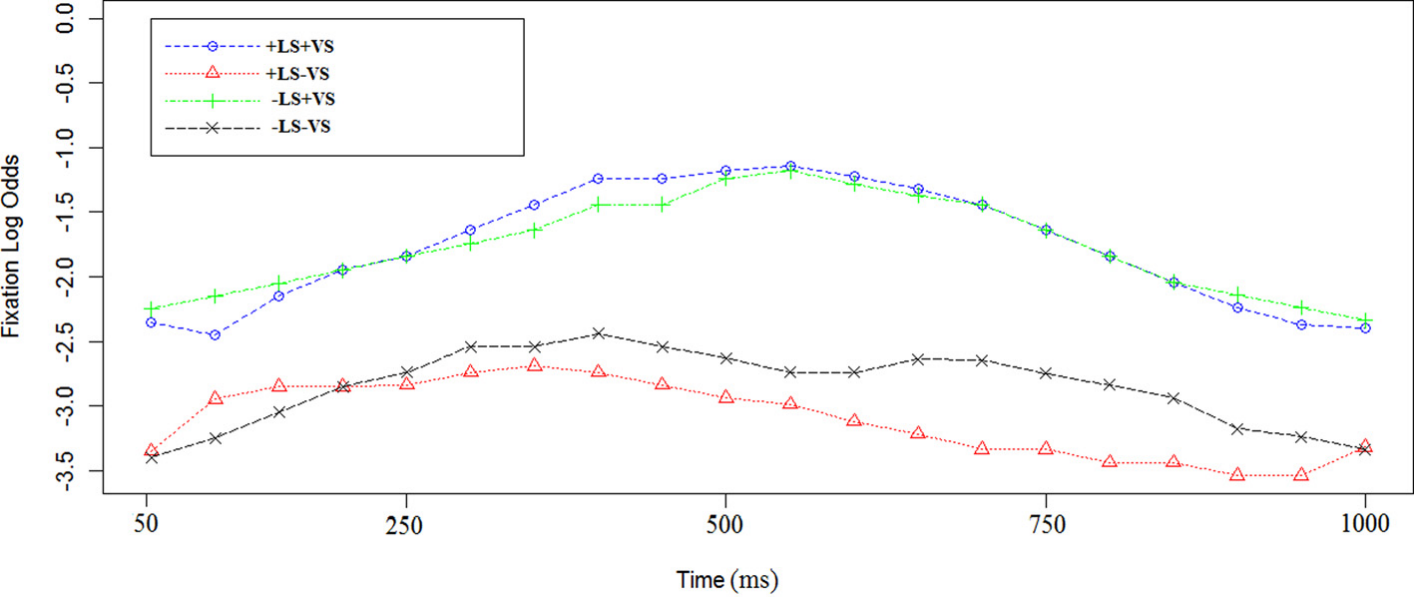
**Log odds of fixations in sentence b. in the four experimental conditions**. In sentence b the entity is not repeated. The logs odds of fixation are referred to the fixations to the sentence target (i.e., the train station). The first time point corresponds to 50 ms after the sentence target onset (train station). Each data point corresponds to 50 ms in the time course.

The time course of log odds of fixations (see Methods) for sentence b. is shown in Figure 3. Sentence b. is the same in all the conditions [see examples (2) and (2′)]. We used a linear mixed-effect regression on the empirical logs of fixations (see Methods for further information). Since no entity had been established as linguistically salient at this point, we found as expected that there was no effect of +LS [Est = 0.5, χ^2^_(1)_ = 1.4, *p* = 0.8].

We did observe an effect of +VS [Est = 2.27, χ^2^_(1)_ = 32, *p* < 0.0002) in sentence b. It is worth noting that the target item of this first instruction was not visually salient. In +VS conditions the only salient landmark on the maps was the target of the last sentence.

Interaction between LS and VS was investigated but not found [Est = 0.2, χ^2^_(1)_ = 1.1, *p* = 0.7].

#### Time to first fixation and fixation dwell

To better understand the influence of visual salience on fixations we compared the time to first fixation to the sentence target (the pictorial landmark of the train station) with respect to all the other landmarks not yet mentioned. Time to first fixation was calculated from the beginning of sentence b. A MANOVA (Null hypothesis tested with Pillai's Trace) found a significant effect for +VS [*Df* = (1, 6), *F* = 4.78, *p* = 0.02]. As expected, no significant difference was found for +LS [*Df* = (1, 6), *F* = 0.46, *p* = 0.49]. No interaction was found between the two conditions [*Df* = (1, 6), *F* = 0.002, *p* = 0.98]. With a visually salient object on the map, the mean time to first fixation for the target was 600 ms from the beginning of the trial, whereas participants looked at the target after 720 ms when there was no visually salient item. The mean time to first fixation of the visually salient item itself was 460 ms compared to 660 ms for that same item when it was not visual salient.

To further explore these effects, we also considered gaze duration (i.e., the total time in ms in which the gaze stayed in the boundaries of the visual target during sentence b.) as a function of visual and linguistic salience with a MANOVA (Null hypothesis tested with Pillai's Trace). Again, we found a significant effect for visual salience [*Df* = (1, 6), *F* = 7.2, *p* = 0.008] but not for linguistic salience [*Df* = (1, 6), *F* = 0.8, *p* = 0.77] and no interaction [*Df* = (1, 6), *F* = 3.8, *p* = 0.06].

Target fixations were longer in the +VS condition (325 ms) and shorter in −VS condition (250 ms). Interestingly, participants tended to fixate the visually salient “distractor” relatively briefly (325 ms) compared to when the last sentence landmark was not visually salient (500 ms).

### Sentence C

#### Log odds of fixations

**Figure 4:**
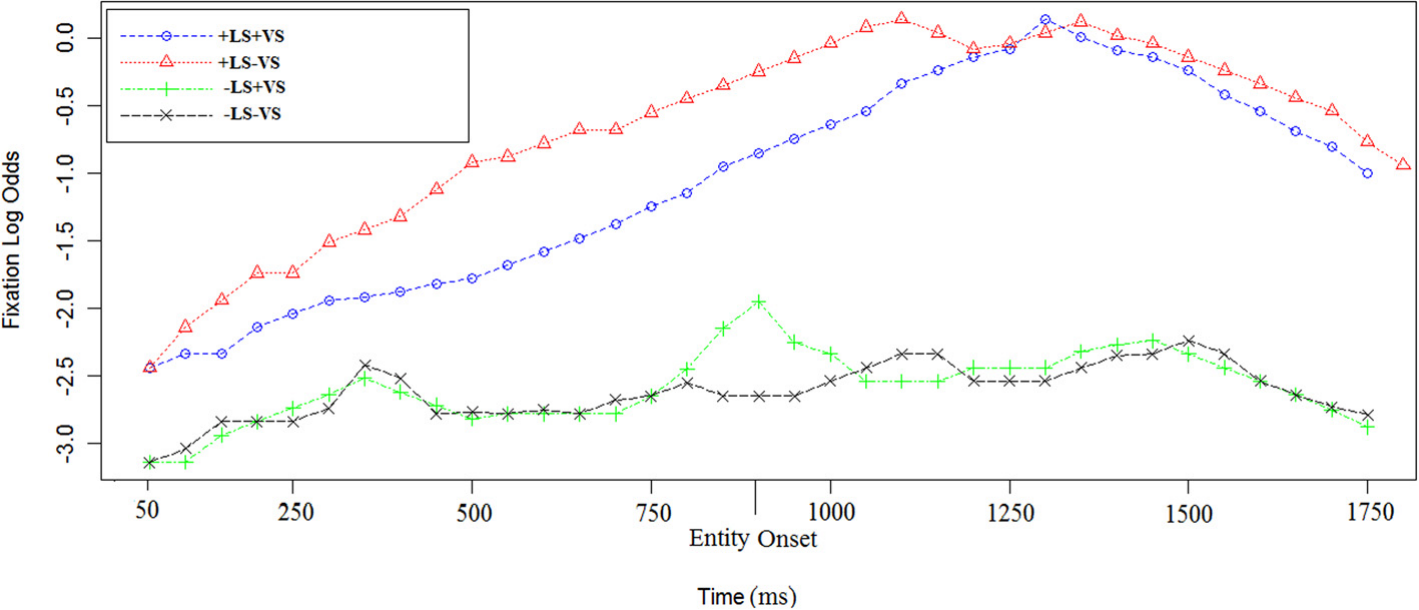
**Log odds of fixations during sentence c. in the four experimental conditions**. The first time point corresponds to 50 ms after the sentence target onset [e.g., “vineyard” in sentence (2)]. The entity onset in +LS condition (e.g., “Depero”) is reported on the graph as well. Each data point corresponds to 50 ms in the time course.

**Figure 5:**
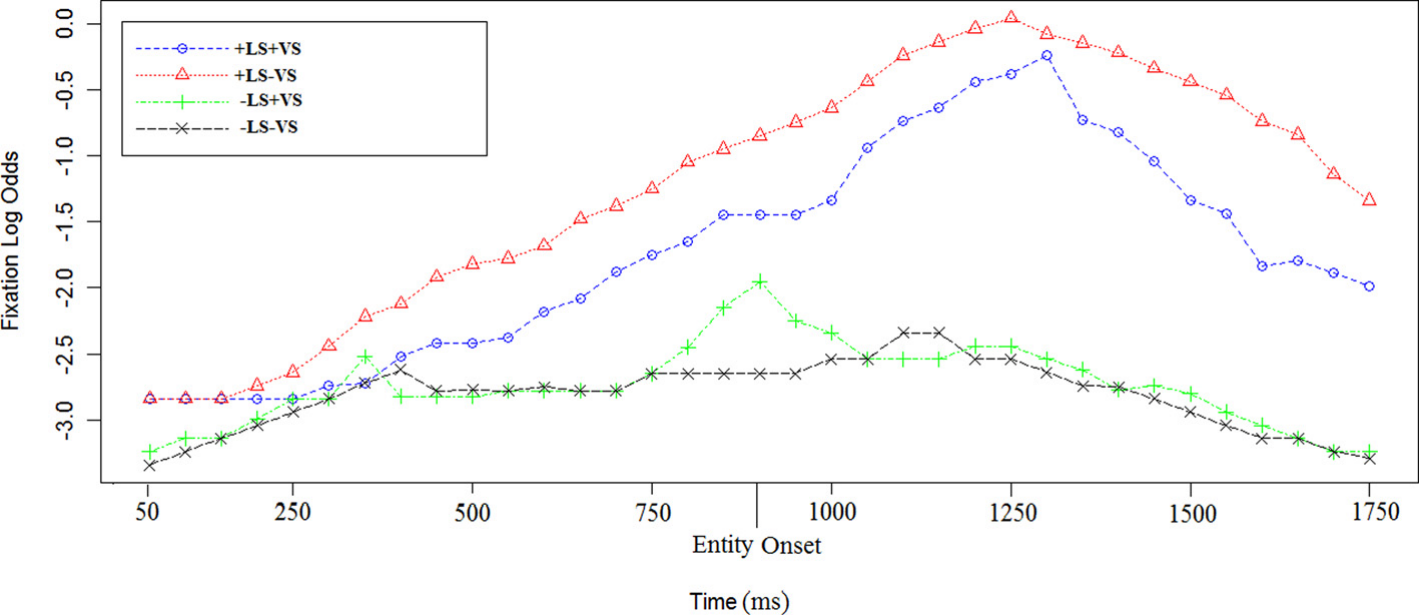
**Log odds of fixations during sentence d. in the four experimental conditions**. The first time point corresponds to 50 ms after the sentence target onset [e.g., “house” in sentence (2)]. The entity onset in +LS condition (e.g., “Depero”) is reported on the graph as well. Each data point corresponds to 50 ms in the time course.

In order to examine the influence of linguistic salience, we examined the time course of fixations in sentence c. and d. in all the visual and linguistic conditions (see Figures 4, 5).

In +LS condition, the first time the entity was heard by the participants was in sentence a., while they attended a fixation cross. In sentence c., participants heard the entity repeated for the second time from the beginning of the trial. For the first time they heard the entity with the actual map in front of them.

The time course of log odds of fixations for sentence c. in (2) and (2′) is shown in Figure 4. Using a linear mixed-effect regression on the empirical logs of fixations we found that there was an effect of +LS [Est = 2.27, χ^2^_(1)_ = 35.1, *p* < 0.0003] and no effect of +VS [Est = 0.1, χ^2^_(1)_ = 1.4, *p* = 0.5]. Further investigations on log odds of fixations before and after entity repetition were run. We found that LS was not significant before the entity repetition [Est = 0.7, χ^2^_(1)_ = 4.8, *p* = 0.1] but it was significant after the entity repetition [Est = 2.1, χ^2^_(1)_ = 26.7, *p* < 0.0001]. Interaction between LS and VS was investigated but not found (Est = 0.1, χ^2^_(1)_ = 0.8, *p* = 0.7).

#### Time to first fixation and fixation dwell

A MANOVA on the time to first fixation at sentence c. target did not find a significant effect for linguistic salience [*Df* = (1, 6), *F* = 0.01, *p* = 0.94; Null hypothesis tested with Pillai's Trace] or for visual salience [*Df* = (1, 6), *F* = 1.95, *p* = 0.16]. The interaction between the two types of salience was not significant as well [*Df* = (1, 6), *F* = 0.98, *p* = 0.32]. Specifically, while in −LS conditions the time to first fixation at the sentence target was 404 ms (−LS+VS) and 480 ms (−LS−VS), in +LS conditions the time to first fixation at the sentence target was 456 ms (+LS−VS) and 521 ms (+LS+VS).

As regards the total duration of fixations at the instruction target, a MANOVA found a significant effect for +LS [*Df* = (1, 6), *F* = 8.8, *p* = 0.003; Null hypothesis tested with Pillai's Trace]. No effect was found for +VS [*Df* = (1, 6), *F* = 0.38, *p* = 0.54] or the interaction between the two factors [*Df* = (1, 6), *F* = 3.48, *p* = 0.06]. The average total fixation duration at sentence c. target was shorter in −LS condition (−LS−VS: 633 ms; −LS+VS: 827 ms) and longer in +LS condition (+LS+VS: 1050 ms; +LS−VS: 1103 ms).

### Sentence D

#### Log odds of fixations

In +LS conditions the entity was repeated in sentence d. The time course of log odds of fixations for sentence d. in (2) and (2′) is shown in Figure 5. Using a linear mixed-effect regression on the empirical logs of fixations we found that there was an effect of +LS [Est = 1.6, χ^2^_(1)_ = 33.3, *p* < 0.0001] and no effect of +VS [Est = 0.03, χ^2^_(1)_ = 1.4, *p* = 0.5]. Interaction were investigated but not found [Est = 0.3, χ^2^_(1)_ = 0.3, *p* = 0.2].

#### Time to first fixation and fixation dwell

A MANOVA on the time to first fixation did not find a significant effect for linguistic salience [*Df* = (1, 6), *F* = 0.1, *p* = 0.74; Null hypothesis tested with Pillai's Trace] or visual salience [*Df* = (1, 6), *F* = 0.55, *p* = 0.46]. The interaction between the two types of salience was not significant either [*Df* = (1, 6), *F* = 2.23, *p* = 0.14]. Specifically, while in the −LS conditions, the time to first fixation was 743 ms (−LS+VS) and 824 ms (−LS−VS), in +LS conditions the target was fixated for the first time after 831 ms (+LS−VS) and 776 ms (+LS+VS) from the beginning of the sentence.

As regards the total duration of fixations at the instruction target, a MANOVA found a significant effect for +LS [*Df* = (1, 6), *F* = 4.1, *p* = 0.04; Null hypothesis tested with Pillai's Trace]. No effect was found for +VS [*Df* = (1, 6), *F* = 1.78, *p* = 0.18], or the interaction between the two salience factors [*Df* = (1, 6), *F* = 0.06, *p* = 0.8]. The average total fixation duration on the target was shorter in −LS condition (−LS−VS: 994 ms; −LS+VS: 1078 ms) and longer in +LS condition (+LS+VS: 1272 ms; +LS−VS: 1160 ms).

### Sentence E

#### Log odds of fixations

**Figure 6:**
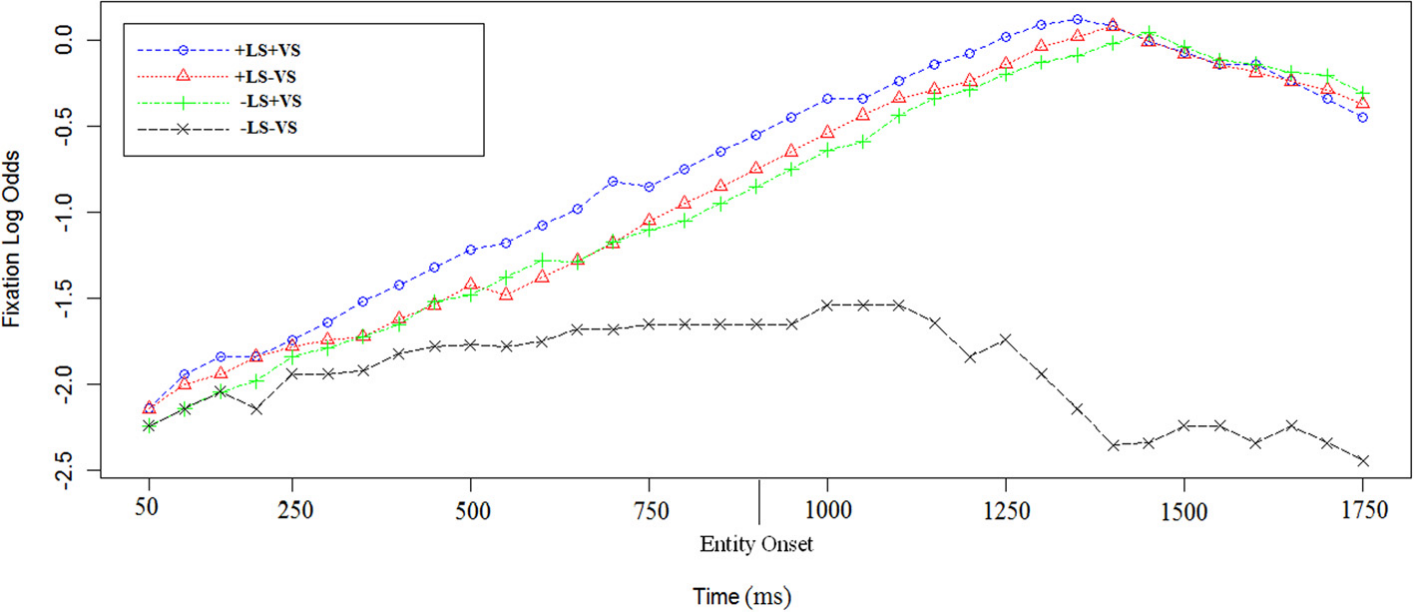
**Log odds of fixations during the last sentence in the four experimental conditions**. In +LS conditions, participants had heard the linguistically salient entity (e.g., “Depero”) repeated for the fourth time. The first time point corresponds to 50 ms after the sentence target onset [e.g., “castle” in sentence (2)]. The entity onset (i.e., “Depero”) is reported on the graph as well. Each data point corresponds to 50 ms in the time course.

Sentence e. is the same in 2 and 2′. A linear mixed-effect regression on the log odds of fixations indicates a significant effect of +LS [Est = 1.15, χ^2^_(1)_ = 26, *p* = 0.0002] and a significant effect of +VS [Est = 1.14, χ^2^_(1)_ = 23.7, *p* = 0.0002; see Figure 6]. Interaction was investigated but not found [Est = 0.1, χ^2^_(1)_ = 0.6, *p* = 0.7].

#### Time to first fixation and fixation dwell

A MANOVA on the time to first fixation on the last sentence target found a significant effect for +LS [*Df* = (1, 6), *F* = 5.13, *p* = 0.03; Null hypothesis tested with Pillai's Trace] but not for +VS [*Df* = (1, 6), *F* =1.1, *p* = 0.3]. We found a partial interaction between the two salience factors [*Df* = (1, 6), *F* =3.9, *p* = 0.05], meaning that, for both +LS and −LS conditions, visual salience slowed down time to first fixation. Specifically, whilst in −LS time to first fixation at the sentence target was respectively 695 (−LS+VS) and +LS+VS (650 ms), it was the shortest in +LS−VS (570 ms) and had an intermediate value in −LS−VS (670 ms). As regards the total duration of fixations to the last sentence target, a MANOVA (Null hypothesis tested with Pillai's Trace) found a significant effect for +VS [*Df* = (1, 6), *F* =6.45, *p* = 0.01] and +LS [*Df* = (1, 6), *F* =12.71, *p* = 0.001], but no interaction between the two factors [*Df* = (1, 6), *F* = 0.44, *p* = 0.5]. The average total fixation duration on the target was the shortest in −LS−VS (700 ms) and the longest in the double salience condition (+LS+VS: 950 ms), with intermediate values for mixed salience conditions (+LS−VS: 850 ms; −LS+VS: 900 ms).

#### Frequency and length of the entity and target words

We checked for frequency and length of target and entity words in the Italian corpus Repubblica (Baroni et al., 2004), consisting of 380.823.725 words. To check that word frequency in sentences b., c., d., and e. did not have an effect on fixations, we added word frequency as a fixed effect in a mixed-effect model with log odds of fixations as dependent variable, by sentence random intercepts and slopes and by subjects random intercepts. No effect of word frequency on log odds of fixations was found [χ^2^_(1)_ = 0.09, *p* = 0.97]. Target and entity words were disyllables and trisyllables. To ensure that word length did not have an effect on fixations, we added word length (number of syllables) as a fixed effect in a mixed-effect model with log odds of fixations as dependent variable, frequency as independent variable, by word length random intercepts and slopes and by subjects random intercepts. No effect of word length on fixations was found [χ^2^_(1)_ = 0.18, *p* = 0.86].

## Discussion

In this study we investigated the gaze pattern during a task in which bottom up visual salience and linguistic salience have been manipulated. The linguistic salient entity was not visually present on the map but only associated with the visual landmarks presented on the map. The hypothesis tested is whether the CF list containing linguistically introduced information and the visual salience map shared the same attentional structure or work concurrently. If only one attentional structure is maintained (i.e., if saliency maps and CF List coincide) we would not expect to observe separate effects of linguistic and visual salience in terms of reading times, fixation dwells and saccadic target selection (time to first fixation). On the other hand, in case the CF List and the saliency map work concurrently, linguistic salience would be expected to affect reading times and fixation dwells but not saccadic target selection.

As regards visual salience, our findings suggest two main effects. First, participants tended to look at the visually salient item when the map appeared in sentence b. Secondly, they were faster, overall, in looking for the correct target of sentence b., having already sampled and excluded the visually salient item in their first fixation(s).

By the time sentence c. was presented to participants, there was no bottom-up effect due to the visually salient landmark, and linguistic salience was the only significant effect. Linguistic salience has an effect on attention after the second repetition of the entity. As for sentence d., there was no bottom up effect of visual salience and time to first fixation was not significant in any of the conditions. With respect to the total duration of fixation, we observed an effect for +LS. Finally, in sentence e. we found a significant effect of both LS and VS on fixations, whereas for the time to first fixation there was a significant effect of +LS but no significant effect of +VS.

Our results show that linguistic salience affects interpretation in terms of reduced response times in a visual world setting as previously reported by, e.g., Arnold et al. (2000). These earlier results have been interpreted as indicating that utterances containing references to linguistically salient entities are easier to process, confirming the predictions of theories of linguistic salience such as Centering (Grosz and Kraus, 1993; Poesio et al., 2004). However, our data show that fixations to visual landmarks increased when these landmarks were associated with a linguistically salient entity that was not present itself on the map. Our finding of increased fixations on a visual target that is merely associated with a linguistically salient entity without actually being the linguistically salient entity is, to our knowledge, entirely novel.

Our second key finding is that although both linguistic and visual salience influenced eye movements and response time in a visual world context, the two salience manipulations affected performance in a different way and appeared to operate independently. Visual salience acted quickly, within the first appearance of the map on the screen, while linguistic salience became significant from sentence c., after the entity repetition was repeated for the second time. The effect of visual salience on fixations during sentence b. could be due to the fact that the visually salient item immediately attracts participants' attention. In the +VS condition, the data from time to first fixation and fixation duration showed that participants quickly fixated, and then discarded, the visually salient item when the map appeared. After that, participants found the task salient item, i.e., the train station, quicker. In maps where all the pictorial landmarks had the same visual salience, this “facilitation” effect was not present.

By the last sentence, linguistic salience influenced both the time course of fixations and response time. In contrast, visual salience influenced the time course of fixations at both the beginning and the end of the trial but did not show a significant effect on response times. With regard to the time to first fixation, when the target landmark was both visually and linguistically salient, it was fixated upon slower and at a similar speed compared to the conditions in which the target was visually salient only or not salient at all. For both +LS and −LS conditions, visual salience slowed down the time to first fixation. These results suggested that the two types of salience work in parallel, slowing down the time to first fixations and lengthening the fixation dwells. That is, when the target landmark was both visually and linguistically salient, it was fixated on for longer, but fixations were quicker when the target item was linguistically salient only. This means that VS no longer had bottom up attention grabbing functions. As +VS slowed the time to first fixation and lengthened fixation dwells, we conclude that the two types of salience are relied on two independent mechanisms. This is corroborated by the results of the response times that are slower in both +VS conditions.

It has been argued that participants already knew the last sentence landmark because of the experimental design. Participants fixated longer the last sentence landmark because they would have learned from previous trials that bigger pictures will be the last sentence target. This explanation, though, does not account for the lack of significance for +VS time to first fixations. If participants had learned that the bigger, colorful landmark was the last sentence target they would have fixated on it before every other target and quicker, but the results for time to first fixation in sentence e. showed that this is not the case. Moreover, if the experimental design would have facilitated the individuation of the last landmark, in +VS conditions participants would have been faster in responding to the last sentence. Again, this is not the case, as the response to visual salient landmarks in the last sentence was significantly slower in +VS than in −VS. +LS conditions were significantly faster than −LS conditions, whereas +VS conditions were not necessarily faster than −VS ones. In +LS and +VS condition, we observed both an increase in fixations on the target related to a linguistically salient entity, and reduced response times. The slowest response times, however, were observed in the −LS+VS condition. This evidence suggests that visual and linguistic salience may involve distinct attentional mechanisms. One possibility is that fixations are driven primarily by a visual-spatial salience map which codes the presence of high priority items in a spatial map. This spatial map integrates both exogenous salience (visual salience, in this case) and endogenous salience based on task relevance; the evidence shown here suggests that the salience map may also be affected by linguistic salience. Salience maps guiding eye movements have been reported mainly in visual and oculomotor areas, suggesting that gaze is closely tied to motor and spatial representations. In contrast, response times would seem to depend on a distinct structure such as the CF list postulated by the Centering Theory. Such a structure affects semantic interpretation and does not integrate visual salience. Under cases of uncertainty or ambiguity, visual information might help to disambiguate the interpretation of the scene, leading to the gaze shifts found in visual world experiments. Likewise, mentioning a word might increase the salience of that item, leading to increased priority for it in the spatial map. Nonetheless, the underlying mechanisms guiding sentence processing (semantic) and gaze control (spatial) may be at least partially dissociable.

### Conflict of interest statement

The authors declare that the research was conducted in the absence of any commercial or financial relationships that could be construed as a potential conflict of interest.

## Appendix

### Map 1

#### +LS+VS

[Oggi visiteremo alcuni luoghi interessanti per studiare la vita del pittore Depero].
Partiamo dalla stazione di Rovereto.
Poi passiamo dal vigneto della famiglia Fedrigotti, per cui Depero ha lavorato molto.
Dopodiché visitiamo la casa dove Depero e' nato.
**Infine andiamo a vedere il Museo Depero al castello**.

#### −LS+VS

[Oggi visiteremo alcune località d'interesse culturale della Val Lagarina].
Partiamo dalla stazione di Rovereto.
Poi passiamo dal vigneto della famiglia Fedrigotti, che possiede una famosa cantina.
Dopodiché visitiamo la casa dove Rosmini e' nato.
**Infine andiamo a vedere il Museo Depero al castello**.

### Map 2

#### +LS−VS

[Oggi andiamo a fare una gita in Val di Fiemme].
Partiamo dalla stazione dei treni.
C'è' una cascata molto bella all'inizio della valle.
C'è' una montagna molto bella continuando lungo la valle.
**C'è un rifugio al fondo della valle**.

#### −LS−VS

[Oggi andiamo a fare una gita].
Partiamo dalla stazione dei treni.
C'è una cascata molto bella all'inizio del percorso.
C'è una montagna molto bella continuando la gita.
**C'è un rifugio al fondo della valle**.

### Map 3

#### +LS+VS

[Oggi andiamo a spasso per Trento].
Partiamo dalla stazione dei treni.
Poi andiamo nella piazza dove gli abitanti di Trento vanno a spasso la sera.
Possiamo andare al bar a bere uno degli spritz migliori di Trento.
**Subito dopo il bar c'è un'altra chiesa, Trento e' una città molto cattolica**.

#### −LS+VS

[Oggi andiamo a spasso per Trento].
Partiamo dalla stazione dei treni.
Poi andiamo nella piazza dove c'è' una antica fontana del Seicento.
Nella piazza possiamo andare al bar a prenderci uno degli spritz migliori della città.
**Subito dopo il bar c'e' un'altra chiesa - Trento è una città molto cattolica**.

### Map 4

#### +LS+VS

[Oggi facciamo una passeggiata per Rovereto].
Partiamo dalla stazione dei treni.
Poi visitiamo il museo di arte contemporanea di Rovereto.
Dopodiché passiamo dalla Piazza principale di Rovereto.
**Infine andiamo a vedere il castello di Rovereto**.

#### −LS+VS

[Oggi facciamo una passeggiata per Rovereto].
Partiamo dalla stazione dei treni.
Poi visitiamo il museo di arte contemporanea del MART.
Dopodiché passiamo dalla piazza principale del Nettuno.
**Infine andiamo a vedere il castello di Rovereto**.

### Map 5

#### +LS+VS

[oggi andiamo a vedere il castello di Rovereto].
Partiamo dalla stazione dei treni.
Sulla collina troveremo il castello.
Attraversando la città vecchia arriviamo al castello.
**Passando per la piazza entriamo nel castello**.

#### −LS−VS

[oggi visitiamo la città di Rovereto].
Partiamo dalla stazione dei treni.
Visitiamo la collina e il suo bosco.
Attraversando la città vecchia arriviamo alla piazza.
**Passando per la piazza entriamo nel castello**

### Map 6

#### +LS+VS

[oggi visitiamo la val di Non].
Partiamo dalla stazione dei treni.
Visitiamo i suoi famosi meleti della valle.
Ora vediamo il lago che taglia la valle.
**Infine visitiamo il monte Roen che chiude la valle**

#### −LS+VS

[oggi visitiamo alcune zone di montagna].
Partiamo dalla stazione dei treni.
Visitiamo i famosi meleti del trentino.
Ora vediamo il lago che è tagliato da una diga.
**Infine visitiamo il monte Roen, che chiude la valle**.

### Map 7

#### +LS+VS

[oggi visiteremo le terme].
Partiamo dalla stazione dei treni.
Attraversando il lago ci avviciniamo alle terme.
Arrivando vicino al monte possiamo vedere le terme.
**Infine arriviamo alle terme**

#### −LS+VS

[oggi vedremo alcune località della Vallagarina].
Partiamo dalla stazione dei treni.
Attraversiamo il lago più famoso della zona.
Arriviamo vicino al monte, il più alto della valle.
**Infine arriviamo alle terme**.

### Map 8

#### +LS−VS

[oggi visiteremo il museo di arte contemporanea di Trento].
Partiamo dalla stazione dei treni.
Dalla collina di Povo il museo è stato trasferito a Trento.
Troviamo un aeroplano nei giardini davanti al museo.
**Infine entriamo al museo di arte contemporanea**.

#### −LS−VS

[oggi visiteremo alcuni musei a Trento].
Partiamo dalla stazione di Trento.
Sulla collina di Povo troviamo il museo della scienza.
Troviamo un aeroplano nei giardini del museo dell'aviazione.
**Infine entriamo al museo di arte contemporanea**.
